# Draft Genome Sequence of *Aquamicrobium defluvii* Strain W13Z1, a Psychrotolerant Halotolerant Hydrocarbon-Degrading Bacterium

**DOI:** 10.1128/genomeA.00984-15

**Published:** 2015-08-27

**Authors:** Xinxin Wang, Decai Jin, Lisha Zhou, Zhuo Zhang

**Affiliations:** a School of Environmental Science and Engineering, Tianjin University, Tianjin, China; b China Offshore Environmental Service Co. Ltd., Tianjin, China; c Research Center for Eco-Environmental Sciences, Chinese Academy of Sciences, Beijing, China; d Shenzhen Key Laboratory of Environmental Microbial Genomics and Application, BGI, Shenzhen, China; e Hunan Plant Protection Institute, Changsha, China

## Abstract

*Aquamicrobium defluvii* W13Z1 was isolated from petroleum-contaminated drill cuttings from the Bohai Sea and could degrade petroleum hydrocarbon with 5% NaCl at 15°C. Here, we present the 4.8-Mb draft genome sequence of this strain, which may provide useful information about the mechanism of petroleum degradation in drill cuttings.

## GENOME ANNOUNCEMENT

Microbial degradation occurs in various hydrocarbon-contaminated environments, such as soil and sediment (1–3). However, little is known about the mechanism of hydrocarbon degradation in drill cuttings, which is a key waste in petroleum production. *Aquamicrobium defluvii* W13Z1, which could degrade petroleum hydrocarbon with 5% NaCl at 15°C, was isolated from petroleum-contaminated drill cuttings from the Bohai Sea (4). However, genomic information about *A. defluvii* is still limited. Here, the draft genome sequence of psychrotolerant halotolerant hydrocarbon-degrading *A. defluvii* W13Z1 is reported for the first time.

Genomic DNA was extracted and sequenced by Illumina HiSeq 2000, which produced 15,519,308 paired-end reads with about 320-fold coverage. Reads were filtered, assembled, scaffolded, gap filled, and validated by NGS QC toolkit version 2.3 (5), SOAPdenovo version 2.04 (6), SSPACE version 2.0 (7), GapFiller version 1.10 (8), and the Burrows-Wheeler alignment tool version 0.7.4 (9). This assembly generated 65 contigs with an *N*_50_ length of 137,118 bp and an average length of 73,452 bp, which were assembled into 59 scaffolds with an *N*_50_ length of 137,153 bp and an average length of 80,925 bp. Genome annotation was carried out using NCBI Prokaryotic Genome Automatic Annotation Pipeline (PGAAP) (http://www.ncbi.nlm.nih.gov/genome/annotation_prok).

The draft genome comprised 4.8 Mb with a GC content of 63.1%. A total of 4,532 coding sequences (CDSs), 45 tRNA genes, 1 noncoding RNA (ncRNA), 1 rRNA operon, and 72 pseudogenes were identified. Of the CDSs, 42.2% can be assigned into 1,911 KEGG orthologous groups by KAAS (10), involving 201 metabolic pathways, and 82.1% can be assigned to COGs with amino acid transport and metabolism as the most abundant class. ISfinder revealed that the IS*481* family dominated the insertion sequence (IS) elements (11). One clustered regularly interspaced short palindromic repeat (CRISPR) element with 20 spacers was detected by CRISPRFinder (12). Plasmid genes essential for stabilization and partition were detected, which suggests the occurrence of plasmid. A total of 142 tandem repeats, 402 potentially secreted proteins, and 7 prophage sequences were identified by tandem repeats finder version 4.08 (13), SignalP version 4.1 (14), and PHAST (15), respectively.

One alkane 1-monooxygenase gene was identified, which was responsible for the initial oxidation of alkanes. Moreover, 10 genes were identified to be involved in the uptake and synthesis of compatible solute, including 7 glycine/betaine ABC transporter genes, 1 ectoine utilization gene, 1 betaine-aldehyde dehydrogenase gene, and 1 alpha,alpha-trehalose-phosphate synthase. Nitrogen fixation genes were detected, which were responsible for the assimilation of N_2_. Moreover, 7 cold shock genes were identified. These genes may be essential to the strain’s survival in a cold saline oligotrophic environment. Information about the genome sequence of *A. defluvii* W13Z1 offered an opportunity to understand genetic diversity of *Aquamicrobium* and the mechanism of hydrocarbon degradation in drill cuttings.

### Nucleotide sequence accession numbers.

The draft genome sequence of *A. defluvii* W13Z1 has been deposited in GenBank under the accession number JENY00000000. The version described in this paper is the first version, JENY01000000.
